# Beneficial Effect of Proline Supplementation on Goat Spermatozoa Quality during Cryopreservation

**DOI:** 10.3390/ani12192626

**Published:** 2022-09-30

**Authors:** Weijing Zhang, Lingjiang Min, Yajing Li, Yaning Lang, S. A. Masudul Hoque, Adedeji Olufemi Adetunji, Zhendong Zhu

**Affiliations:** 1 College of Animal Science and Technology, Qingdao Agricultural University, Qingdao 266109, China; 2 Department of Animal Breeding and Genetics, Faculty of Veterinary Medicine and Animal Science, Bangabandhu Sheikh Mujibur Rahman Agricultural University, Gazipur 1706, Bangladesh; 3 Department of Animal Sciences, North Carolina Agricultural and Technical State University, Greensboro, NC 27411, USA

**Keywords:** cryopreservation, goat sperm, proline, quality, antioxidant

## Abstract

**Simple Summary:**

Sperm suffers damage from reactive oxygen species stress (ROS) during cryopreservation. Supplementation of antioxidants is suggested to reduce sperm cryodamage induced by ROS. Proline has been regarded as a powerful antioxidant. However, there is no information on the role proline plays during goat sperm cryopreservation. Thus, this study aims to investigate the effects of proline during goat sperm cryopreservation procedures. In the present study, supplementation of 2 mM proline significantly enhanced post-thaw goat sperm quality by regulating sperm redox homeostasis. Proline supplementation protects goat sperm against ROS stress through PRODH-mediated metabolism.

**Abstract:**

Sperm cryopreservation contributes to the extensive utilization of artificial insemination (AI) in the daily livestock industry. However, due to the presence of few sperm with good biological function in post-thaw goat sperm, its use has been limited for AI purposes. Hence, its improvement has been the focus of many research studies. This study aimed to investigate the effects of proline supplementation of the freezing medium on goat sperm. The goat semen was cryopreserved with freezing medium supplementation of different concentrations of proline (0, 0.5, 1, 2 and 4 mM). The post-thaw sperm motility patterns, membrane integrity, acrosome integrity, lipid peroxidation (LPO) levels, malondialdehyde (MDA) levels, total antioxidant capacity (T-AOC), proline dehydrogenase (PRODH) activity, superoxide dis-mutase (SOD) activity, glutathione (GSH) levels and GSH/GSSG were evaluated. Likewise, the expression and immunofluorescent localization of PRODH in post-thaw goat sperm was also detected. It was observed that addition of 2 mM proline to the freezing medium significantly enhanced post-thaw goat sperm total motility, progressive motility, straight-linear velocity (VSL), curvilinear velocity (VCL), average path velocity (VAP), straightness (STR), linearity (LIN), membrane integrity and acrosome integrity. Interestingly, PRODH was expressed in post-thaw goat sperm, especially in the post-acrosome and sperm tail. Addition of 2 mM proline also significantly increased the post-thaw sperm PRODH activity compared to the control. Moreover, post-thaw goat sperm LPO levels and MDA levels were reduced by supplementation of 2 mM proline. Furthermore, compared to the control, the values of post-thaw goat sperm T-AOC, SOD activity, GSH level and GSH/GSSG were also significantly increased in 2 mM proline treatment. Reduction of post-thaw goat sperm apoptosis in 2 mM proline treatment was also observed as the levels of Caspase3 and Caspase9 were decreased by the supplementation with 2 mM proline. These observations suggest that the addition of 2 mM proline to the freezing medium increased post-thaw goat sperm quality by reducing oxidative stress during cryopreservation. These findings also provide novel insights into the use of proline as an efficient additive to enhance post-thaw goat sperm quality during cryopreservation.

## 1. Introduction

Sperm cryopreservation technology greatly contributes to preservation of genetic materials, especially for endangered species, and promotes the use of artificial insemination in domestic animals [1]. However, during the cryopreservation process, sperm suffer irreversible damage due to cold shocks [2], osmotic stresses [3,4,5] and oxidative stresses [6], which lead to reduced sperm fertilization capability [7]. Despite 40–60% of post-thaw goat sperm being motile, less than 30% are biologically functional [8]. Recently, some researchers developed a freezing-thawing process that improves the post-thaw goat sperm quality, but the procedure does not align with the accepted optimum cryopreservation protocol for goat semen [9,10,11]. Hence, novel strategies aimed at improving goat sperm cryopreservation will help in increasing post-thaw sperm quality and in accelerating large-scale goat production.

Previous studies reported that freezing-thawing processes were associated with damaged cellular membranes [12], changed the stability of the lipid bilayer as well as surface proteins of the membrane [13], interrupted cellular metabolism homeostasis [14], damaged DNA integrity [15] and reduced sperm motility [16]. Most of the aforementioned sperm damage is caused by ice crystal formation and the overproduction of reactive oxygen species (ROS) that occur during cryopreservation [17]. In goat sperm, the plasma membrane contains a large amount of polyunsaturated fatty acids that are affected by ROS accumulation [18]. To maintain sperm quality during the cryopreservation process, it is important to balance the production of ROS and its scavenging. A major strategy for enhancing goat sperm’s resistance to oxidative stress during cryopreservation is the supplementation of exogenous antioxidants in the freezing medium, such as glutathione [19], green synthesized anionic cupric oxide nanoparticles [20], Lycium barbarum and Laminaria japonica polysaccharides [21], turraea fischeri leaf extract [22], resveratrol and taurine [23], vitamin E and amino acids [24], trehalose [25], myo-inositol and melatonin [26]. However, most of the exogenous antioxidants are unstable due to oxidation; hence, they lose their anti-oxidative capacity. 

Recently, proline, a multi-functional amino acid, has been shown to function in osmotic and oxidative stress protection, carbon and nitrogen metabolism, as well as cell signaling and cell survival [27,28]. Several reports revealed that proline could protect fungi, plants and mammalian cells from oxidative damage because of its antioxidant effects [29,30]. When HEK293 cells were treated with different oxidative stress inducers, cellular proline biosynthesis was found to be upregulated in a bid to suppress cell apoptosis by modulating intracellular redox [29]. In addition, under exposure to hydrogen peroxide, treatment of Wistar melanoma 35 cells with proline improved cell viability, reduced oxidative damage of cellular lipids and proteins, and retained ATP and NADPH levels via proline dehydrogenase activity [31]. Moreover, proline has also been used as an additive cryoprotectant during cell cryopreservation, such as in ram sperm [32], Andalusian donkeys’ sperm [33], mice oocytes [34] and in human endothelial cells [35]. In our previous study, we showed that boar sperm expressed proline dehydrogenase, and that proline increased the activity of proline dehydrogenase and improved boar sperm quality during storage [36]. However, for goat sperm cryopreservation regimes, the cryoprotective effects of proline on goat sperm have not been reported. Furthermore, with numerous studies reporting that proline protection involves protein chaperoning, direct scavenging of ROS, protection and upregulation of antioxidant enzymes, and maintenance of glutathione and NADPH/NADP^+^, it is imperative to understand the mechanism of proline protection. Therefore, this study evaluates the effect of proline on goat sperm cryotolerance, and provides a novel insight into proline protection mechanisms against ROS by the detection of proline metabolic enzymes.

## 2. Materials and Methods

### 2.1. Chemicals 

Unless stated specially, all chemicals were purchased from Sigma-Aldrich (Shanghai, China).

### 2.2. Ethical Approval 

All animals and experimental procedures were approved by the Qingdao Agriculture University Institutional Animal Care and Use Committee (QAU1121010). 

### 2.3. Animals and Semen Collection

Five fertile and healthy Laoshan bucks aged between 1.5 and 2 years were used in this study. The bucks were housed individually, fed basal diets and provided free access to water. Bucks’ semen was collected weekly for five successive weeks using an artificial vagina. A total of 25 ejaculates from five bucks were used in the present study. Ejaculates from each buck were kept separately and placed in a 35 °C incubator during transport to the laboratory. The motility of ejaculated semen was assessed using the computer-assisted sperm analysis (CASA) system, and only semen with more than 80% motility was used. The ejaculated semen from five bucks in each replicate was pooled to minimize individual differences, split into 5 parts and cryopreserved in freezing medium supplemented with different concentrations of proline (0, 0.5, 1, 2 and 4 mM). 

### 2.4. Semen Freezing and Thawing

The freezing extender was composed of 250 mM Tris, 83 mM citric acid, 69 mM fructose, 5% (*v*/*v*) glycerol and 20% (*v*/*v*) egg yolk. The semen samples were diluted with freezing extenders that contain different doses of proline (0, 0.5, 1, 2 and 4 mM) at a concentration of 1 × 10^8^ spermatozoa/mL, and then cooled to 4 °C for 2 h. The cooled diluted samples were loaded into 0.25 mL plastic straws. Then, straws were placed horizontally 10 cm above liquid nitrogen (LN) for 10 min, before storage in LN. After one week, the frozen semen was thawed to evaluate sperm quality. During the thawing process, the frozen straws were placed in a 37 °C water bath for 30 s. 

### 2.5. Evaluation of Sperm Motility

Sperm motility was measured with the computer-assisted sperm analysis (CASA) system (Nikon, ECLIPSE E200, Shanghai, China). Briefly, a drop of 6 μL post-thaw sperm sample was placed on a glass slide, and in each treatment three fields were randomly selected for evaluating motility patterns. Total motility was defined as the percentage of motile sperm moving with a path velocity >12 μm/s. Progressive motility was defined as the percentage of motile sperm moving with a path velocity 45 μm/s and in a straight line for over 80% of the time.

### 2.6. Evaluation of Membrane Integrity and Acrosome Integrity

According to our previous studies [37,38,39], the LIVE/DEAD™ Sperm Viability Kit (Invitrogen™, Shanghai, China) and fluorescent isothiocyanate-peanut agglutinin (FITC-PNA, Sigma-Aldrich, Shanghai, China) were used for the evaluation of sperm membrane integrity and acrosome integrity, respectively. Briefly, for membrane integrity evaluation, sperm was incubated with SYBR-14 working solution and PI stocking solution in the dark. The stained sperm were observed and photographed using an epifluorescence microscope (ZEISS DM200LED, Oberkochen, Germany) with 488 nm excitation for SYBR-14 green fluorescence and 535 nm excitation for PI red fluorescence.

In terms of acrosome integrity detection, the sperm was incubated with 100 μg/mL fluorescein isothiocyanate-peanut agglutinin solution for 30 min after fixing, then incubated with 2.4 mM PI stocking solution for another 10 min. Stained sperm samples were observed and photographed with an epifluorescence microscope (ZEISS DM200LED, Oberkochen, Germany) with 488 nm excitation for FITC-PNA green fluorescence and 535 nm excitation for PI red fluorescence.

### 2.7. Lipid Peroxidation

Lipid peroxidation assay kit (A106-1-1, Nanjing Jiancheng Bioengineering Institute, Nanjing, China) was used to evaluate sperm lipid peroxidation. Briefly, sperm samples were centrifuged at 2500× *g* after ultrasonication on ice. Then, the supernatant was collected and mixed with application solution I and application solution II from the kit, and incubated at 45 °C for 60 min for the detection of lipid peroxidation. The absorbance readings were carried out using a microplate reader (TECAN, Infinite M Nano, Männedorf, Switzerland) at 586 nm according to the manufacturer’s instructions.

### 2.8. Measurement of Post-Thaw Sperm Malondialdehyde Content

Sperm malondialdehyde (MDA) content was measured with MDA assay kit (A003-1-2, Nanjing Jiancheng Bioengineering Institute, Nanjing, China). Briefly, the post-thaw sperm were ultrasonicated (20 kHz, 300 W, operating at 50%, 3 min for 10 s on and 5 s off) on ice. The sample was mixed with the pre-prepared reaction buffer reagent, boiled for 40 min, then centrifuged for collecting supernatant after cooling. The absorbance was measured at 532 nm with a microplate reader (TECAN, Infinite M Nano, Männedorf, Switzerland).

### 2.9. Measurement of Superoxide Dismutase Activity

Total superoxide dismutase (SOD) assay kit (A001-3-2, Nanjing Jiancheng Bioengineering Institute) was used to detect the activity of SOD. Briefly, sperm samples were centrifuged at 3000× *g* for 10 min after ultrasonication (20 kHz, 300 W, operating at 50%, 5 cycles for 5 s on and 30 s off) on ice. Then, the supernatant was collected and a enzyme working solution and substrate solution were added to it before incubation, followed by incubation at 37 °C for 20 min. The absorbance readings were carried out using a microplate reader (TECAN, Infinite M Nano, Männedorf, Switzerland) at 450 nm.

### 2.10. Measurement of Glutathione (Reduced) and Glutathione (Oxidized) Levels

Total glutathione (T-GSH) levels and glutathione (Oxidized) (GSSG) levels were evaluated with Total glutathione/Oxidized glutathione assay kit (A061-1-2, Nanjing Jiancheng Bioengineering Institute). Sperm samples were added to working solution IV and centrifuged at 3500× *g* for 10 min after ultrasonication on ice. For the detection of sperm T-GSH content, working solutions I, II, III were added to the supernatant sample, and the absorbance was measured with a microplate reader (TECAN, Infinite M Nano, Männedorf, Switzerland) at 405 nm at 30 s and 10 min 30 s points. For measuring the GSSG content, supernatants were pretreated with working solutions V and VI, and incubated at 37 °C for 30 min; the subsequent processes were the same as for T-GSH content detection. GSH content was calculated according to the formula: GSH = T − GSH − 2 × GSSG.

### 2.11. Measurement of Total Antioxidant Capacity

Total antioxidant capacity assay kit (A015-2-1, Nanjing Jiancheng Bioengineering Institute, Nanjing, China) was used to detect the sperm antioxidant capacity. Briefly, the samples were lysed and centrifuged to collect the supernatant. The supernatant was mixed with ABTS working solution and absorbance readings were carried out using a microplate reader (TECAN, Infinite M Nano, Männedorf, Switzerland) at 410 nm.

### 2.12. Western Blotting Analysis

Sperm total protein was extracted with sodium dodecyl sulfate (SDS) sample buffer. The total protein (20 µg) of each sample was separated by 10% SDS-PAGE gel (EC0023-B, Sparkjade, Jinan, China) and transferred onto a polyvinylidene fluoride (PVDF) membrane. The non-specific bindings of PVDF membranes were blocked with 5% (*m*/*v*) bovine serum albumin diluted with TBST (1% TBS, 0.1% Tween 20). These membranes were then immunoblotted with diluted primary antibodies against PRODH (22980-1-AP, 1:3000, Proteintech, Wuhan, China), Caspase3(A2156, 1:1000, AB clonal, Wuhan, China), Caspase9 (A0281, 1:1000, AB clonal, Wuhan, China) or tubulin (AC008, 1:1000, AB clonal, Wuhan, China) in TBST solution (1:1000 final dilution) and incubated at 4 °C for 12 h. Subsequently, membranes were washed with TBST solution and incubated with secondary antibodies (AS014, 1:1000, AB clonal, Wuhan, China) for 2 h, and then washed with TBST solution for three times. The ECL plus (ED0016-B, Sparkjade, Jinan, China) was used for detection and developed by gel imaging analyzer (Alpha, Fluor Chem Q, Shanghai, China). 

### 2.13. Immunofluorescence 

Sperm samples were washed with PBS after being fixed, and permeabilized with 3% (*v*/*v*) Triton X-100 in 1× PBS for 1 h. Subsequently, sperm sample was blocked with 10% goat serum (*v*/*v*) at room temperature for 30 min, and incubated with a primary antibody against PRODH (22980-1-AP, 1:200, Proteintech, Wuhan, China) at 4 °C overnight. The sperm samples were incubated with fluorescent secondary antibody (Cy3-labeled Goat Anti-Rabbit IgG, 1:200; Beyotime Institute of Biotechnology, A0516) in the dark after washing with PBS containing 1% BSA. DAPI (2-(4-Amidinophenyl)-6-indolecarbamidine dihydrochloride, 1:1000; Beyotime Institute of Biotechnology, C1002) was used to stain sperm nuclear contents. The stained sperm were observed with a fluorescence microscope (ZEISS, DM200LED, Oberkochen, Germany).

### 2.14. Evaluation of Proline Dehydrogenase Activity 

A proline dehydrogenase (PRODH) assay kit (BC4160, Beijing Solarbio Science & Technology Co., Ltd., Beijing, China) was used to evaluate the activity of PRODH in goat sperm based on the manufacturer’s instructions. In brief, sperm samples were lysed, centrifuged and the supernatant was collected. Subsequently, the supernatant was mixed with the reaction solution. The absorbance readings were carried out using a spectrophotometer (Yoke Instrument Co., Ltd., UV755B, Shanghai, China) at 600 nm to evaluate sperm PRODH activity. 

### 2.15. Statistical Analysis

Data from three replicates were compared using either Student’s *t*-test or one-way analysis of variance followed by Tukey’s post hoc test (Statview; Abacus Concepts, Inc., Berkeley, CA, USA). All values are presented as the mean ± standard deviation (SD). Differences between treatments were considered statistically significant at *p* < 0.05.

## 3. Results

### 3.1. Proline Affected the Post-Thaw Sperm Motility Parameters

As shown in Table 1, post-thaw goat sperm motility data analyzed by CASA show that proline addition significantly (*p* < 0.05) increased parameters such as sperm total motility (TM), progressive motility (PM), curvilinear velocity (VCL), average path velocity (VAP), straightness (STR), linearity (LIN), and straight-line velocity (VSL), compared to the control. The supplementation of the freezing medium with 2 mM proline gave the highest value for post-thaw goat sperm motility parameters among all treatments (*p* < 0.05). Interestingly, proline addition had no significant influence on parameters such as beat-cross frequency (BCF), post-thaw goat sperm lateral head (ALH), and wobble (WOB), as their values were similar to the control group.

### 3.2. Proline Affected Post-Thaw Goat Sperm Membrane Integrity and Acrosome Integrity

**Figure 1:**
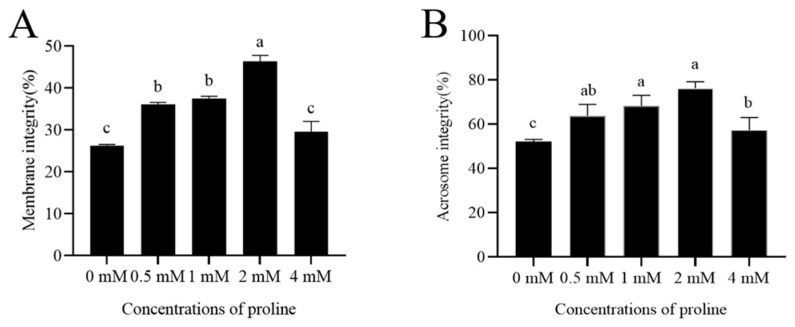
Detection of post-thaw goat sperm membrane integrity and acrosome integrity. (**A**) Effects of different concentrations of proline on post-thaw goat sperm membrane integrity. (**B**) Effects of different concentrations of proline on post-thaw goat sperm acrosome integrity. Values are specified as mean ± standard deviation (SD). Columns with different lowercase letters differ significantly (*p* < 0.05). Bars = 30 μm, *n* = 5.

The addition of proline from 0.5 mM to 2 mM significantly increased the post-thaw goat sperm membrane integrity when compared to the control (*p* < 0.05). The percentage of post-thaw sperm membrane integrity in the 2 mM proline treatment was the highest (Figure 1A). However, values for control and 4 mM proline treatments were similar for post-thaw sperm membrane integrity.

Interestingly, it was observed that the addition of proline from 0.5 mM to 4 mM to the freezing medium greatly contributed to maintaining post-thaw goat sperm intact acrosomes (*p* < 0.05); moreover, the 2 mM proline treatment showed the highest value of intact acrosomes among the treatments (Figure 1B).

### 3.3. Proline Reduced the Post-Thaw Sperm Oxidative Stress via Enhancing Its Anti-Oxidative Ability

**Figure 2:**
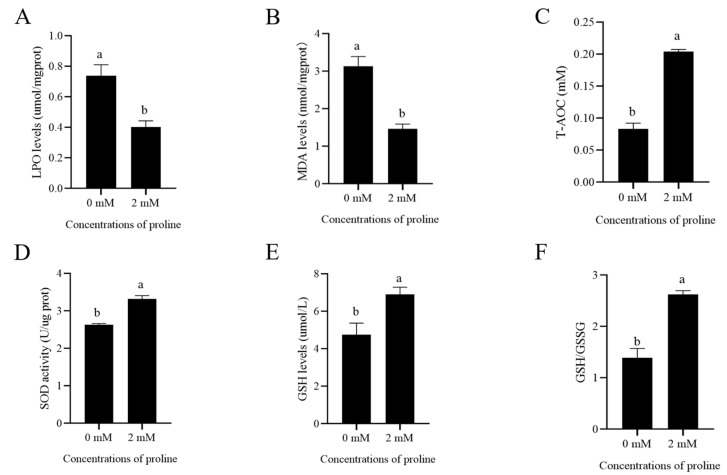
Effects of supplementation of the freezing medium with 2 mM proline on post-thaw goat sperm LPO level (**A**), MDA levels (**B**), T-AOC (**C**), SOD activity (**D**), GSH levels (**E**), and ratio of GSH/GSSG (**F**). Values are specified as mean ± standard deviation (SD). Columns with different lowercase letters differ significantly (*p* < 0.05). LPO, lipid peroxidation; MDA, malondialdehyde; T-AOC, total antioxidant capacity; SOD, superoxide dismutase; GSH, reduced glutathione; GSSG, oxidized glutathione, *n* = 5.

As shown in Figure 2A,B, supplementation of 2 mM proline to the freezing medium significantly decreased the post-thaw goat sperm LPO levels and MDA levels compared to the control (*p* < 0.05). When the post-thaw sperm T-AOC was evaluated, the addition of 2 mM proline to the freezing medium also significantly enhanced sperm T-AOC (Figure 2C, *p* < 0.05). Similarly, SOD activity and GSH levels in 2 mM proline treatment were much higher than in the control (Figure 2D,E, *p* < 0.05). Moreover, addition of 2 mM proline to the freezing medium also significantly increased the GSH/GSSG levels (Figure 2F, *p* < 0.05). Those data indicated that the post-thaw goat sperm’s anti-oxidative stress was enhanced by proline addition to the freezing medium.

### 3.4. Proline Protected Sperm against Antioxidative Stress via Proline Dehydrogenase

**Figure 3:**
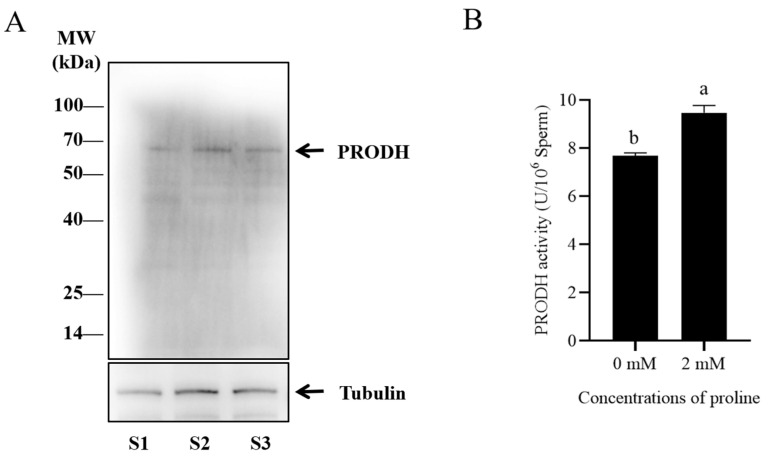
(**A**) Western blotting analysis of PRODH in goat sperm. (**B**) Effects of proline on PRODH activity in post-thaw goat sperm. Values are specified as mean ± standard deviation (SD). Columns with different lowercase letters differ significantly (*p* < 0.05). PRODH: proline dehydrogenase; S1: goat sperm sample 1; S2: goat sperm sample 2; S3: goat sperm sample 3. Bars = 30 μm, *n* = 3.

**Figure 4:**
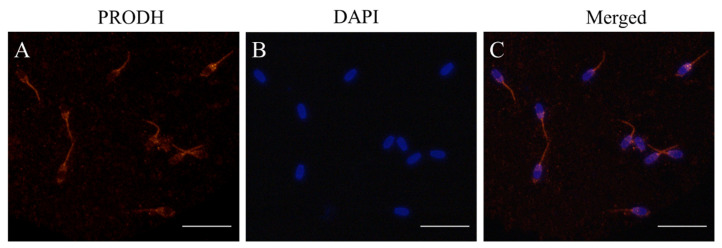
(**A**–**C**) Detection and immunofluorescent localization of PRODH in post-thaw goat sperm. (**B**) The sperm nuclear contents were stained with DAPI; sperm with red fluorescence indicated that the PRODH was expressed in the post-thaw acrosome and sperm tail.

Western blot and immunofluorescence methods were used to detect the presence of PRODH in post-thaw goat sperm using a specific antibody. It was observed that the bank of 68 kDa representing the PRODH protein was present in goat sperm (Figure 3A and Figure S1). As shown in Figure 4, the PRODH was expressed in the post-thaw acrosome and sperm tail. When the PRODH activity in the post-thaw goat sperm was measured, supplementation of 2 mM proline significantly increased it compared to the level in the control (Figure 3B).

### 3.5. Proline Decreased the Post-Thaw Sperm Apoptosis 

**Figure 5:**
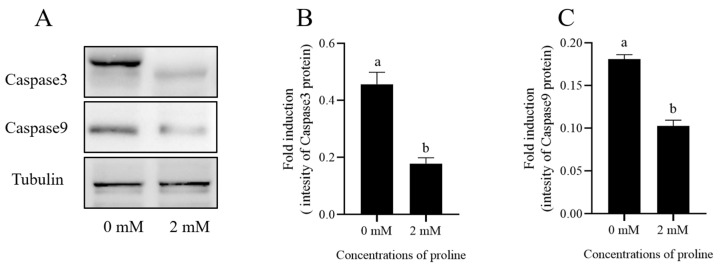
(**A**) Effects of 2 mM proline on protein (Caspase3 and Caspase9) expression in post-thaw goat sperm were detected by Western blotting. (**B**,**C**) The intensity of the bands was analyzed using a Gel-Pro Analyzer (Media Cybernetics, MD, USA). Values are means ± standard deviation (SD) of 3 replicates. Columns with different lowercase letters differ significantly (*p* < 0.05), *n* = 3.

Post-thaw goat sperm apoptosis was evaluated by the Western blot method, and it was observed that the addition of 2 mM proline to the freezing medium significantly decreased the levels of Caspase3 and Caspase9 in the post-thaw goat sperm when compared to the control (*p* < 0.05) (Figure 5 and Figure S2).

## 4. Discussion

Post-thaw goat sperm with good quality is very important for the effectiveness of artificial insemination using frozen semen [40]. The present study aimed to investigate whether supplementation of the freezing medium with proline improves goat frozen-thawed sperm, and how proline protects sperm from cryo-damage during cryopreservation. We showed that the addition of 2 mM proline to the freezing medium significantly improved post-thaw sperm motility parameters, membrane integrity, acrosome integrity and T-AOC, SOD activity, GSH level, and GSH/GSSG, while decreasing the sperm LPO, MDA levels and apoptosis.

ROS are generated in sperm during the cooling, freezing and thawing processes [41]. When ROS production exceeds goat sperm’s ROS scavenging ability, sperm are subjected to oxidative stress and their functioning deteriorates [42]. It is known that sperm membrane contains a high amount of polyunsaturated fatty acids (PUFA) [43]. The structure of PUFA is susceptible to ROS induced damage and the initiation of LPO leads to a change in sperm membrane composition and biophysical properties [18]. Although the goat sperm and seminal plasma could overcome some degree of ROS stress because they possess an antioxidant defense system, this capacity is limited since many of the cytoplasmic components are lost during spermiogenesis [44]. Thus, goat sperm are sensitive to ROS stress during the cryopreservation process, and the supplementation of freezing mediums with antioxidants would be beneficial to improving sperm quality. In this study, the addition of proline improved the post-thaw goat sperm motility parameters during cryopreservation, and this is in consonance with findings of a study on donkey sperm [45]. Oxidative, thermal, and osmotic stresses damage the sperm membrane and acrosome during cryopreservation. However, the supplementation of proline improved goat sperm membrane integrity and acrosome integrity, which is in agreement with previous studies conducted on donkeys [45], cynomolgus monkeys [46], bulls [47] and humans [48]. However, those results disagreed with Ollero et al. (1998) [49] who found that addition of proline did not improve post-thaw ram sperm membrane integrity and acrosome integrity under cryopreserved sperm using Fisher’s medium condition. The difference in results might be a result of the use of different extenders. Our results suggest that supplementing proline in freezing mediums enhances the structural integrity of goat spermatozoa after cryopreservation.

Moreover, the present study reported that supplementation of proline decreased the LPO and MDA levels, but increased the T-AOC level, SOD activity, and GSH level. These results align with previous studies which demonstrated that sperm ROS damage was reduced by addition of proline to increase the sperm antioxidative ability [36,48,50]. Osmotic stress was reported to be correlated with the overproduction of ROS [51], and this condition occurs during cryopreservation, disrupting the mitochondrial membrane within dehydration-rehydration cycles [52]. As seen in this study, freezing the sperm with proline, a notable permeable cryoprotectant, helped in decreasing the osmotic stress in sperm, thus suppressing ROS generation and reducing reduce the post-thaw sperm LPO and MDA levels. In addition, we found that the goat sperm T-AOC and SOD activity was increased by the addition of proline. These findings agreed with Zarse et al. (2012) [53], who found that catabolism of proline was purported to increase worms’ antioxidant enzymes expression via activated homologues of p38 MAP kinase and that nuclear factor erythroid 2-related factor 2 regulates the cellular redox homeostasis. 

The key feature of proline by which it quenches singlet oxygen in somatic cells is the secondary amine structure of pyrrolidine [54]. In our previous study, we showed that the exposure of boar sperm to DL-pipecolinic acid shared secondary amine with proline improved sperm progressive motility; however, the THFA failed to do so, because lacks a secondary amine. Therefore, it is noteworthy that the antioxidative effect which proline exhibits in sperm is as a result of its secondary amine structure [36]. Proline dehydrogenase is an oxidoreductase enzyme that catalyzes proline for redox. Proline dehydrogenase mainly catalyzes the dehydrogenation of the ketone group (-RCO) and alkyl group (-CH2-CH2-) of proline. After dehydrogenation, proline forms P5C, which is then converted to glutamic acid or reduced to proline under the action of P5CDH or P5CR, respectively, with either NADH or NADPH as cofactors [36]. In this study, we demonstrated that the PRODH was expressed in the post-thaw goat sperm post-acrosome and the tail. The location of PRODH in post-thaw goat sperm was quite different from its location in boar sperm [36]; this might be due to the different species. Interestingly, our results show that PRODH activity in post-thaw goat sperm was enhanced with proline addition during cryopreservation. Thus, PRODH is involved in proline’s characteristic protective role in post-thaw goat sperm.

In addition, when post-thaw goat sperm apoptosis was evaluated in this study, we observed that the levels of caspase 3 and caspase 9 in post-thaw goat sperm decreased significantly with the addition of proline to the freezing medium. GSH/GSSG is one of the key factors for regulating the mitochondrial permeability transition pore (PTP). The reduction of the GSH/GSSG would make PTPs open, and initiate the release of apoptosis initiating factors which include cytochrome C, apoptotic protease activator and apoptosis-inducing factor from mitochondria, then activating caspase 9 and caspase 3 [55,56]. Thus, supplementation of proline might reduce sperm apoptosis via improving the GSH/GSSG level to protect mitochondria in post-thaw sperm.

## 5. Conclusions 

Supplementation of proline in the freezing medium enhanced post-thaw goat sperm quality by regulation of the sperm redox homeostasis. Proline protects goat sperm against ROS stress via the PRODH-mediated metabolism. Overall, our study provides a novel insight into the use of proline in sperm supplementation to enhance post-thaw goat sperm quality during cryopreservation.

## Figures and Tables

**Table 1:** Effect of proline on post-thaw goat sperm motility parameters measured with CASA.

Sperm Parameters	0 mM	0.5 mM	1 mM	2 mM	4 mM
Total motility (%)	37.93 ± 0.67 ^b^	38.63 ± 1.19 ^b^	45.07 ± 2.31 ^b^	52.55 ± 4.60 ^a^	40.78 ± 1.42 ^c^
Progressive motility (%)	25.57 ± 2.65 ^c^	28.97 ± 2.33 ^b^	33.43 ± 2.16 ^ab^	36.03 ± 1.23 ^a^	30.43 ± 1.34 ^c^
VCL (μm/s)	41.88 ± 5.09 ^b^	60.17 ± 5.36 ^a^	58.36 ± 5.26 ^a^	61.29 ± 6.38 ^a^	40.69 ± 4.59 ^b^
VSL (μm/s)	30.37 ± 1.46 ^c^	44.36 ± 1.17 ^b^	46.34 ± 5.22 ^b^	59.54 ± 5.93 ^a^	41.17 ± 1.32 ^b^
VAP (μm/s)	33.99 ± 2.53 ^bc^	42.68 ± 2.59 ^ab^	43.07 ± 3.79 ^ab^	48.72 ± 2.29 ^a^	37.02 ± 2.15 ^b^
BCF (Hz)	8.35 ± 0.17	9.79 ± 1.30	7.48 ± 0.92	8.75 ± 0.44	9.02 ± 0.85
ALH (μm)	2.83 ± 0.34	2.81 ± 0.37	3.43 ± 0.44	3.90 ± 0.34	3.40 ± 0.22
STR (%)	75.46 ± 4.79 ^b^	85.83 ± 1.74 ^a^	81.33 ± 2.59 ^a^	82.60 ± 1.96 ^a^	80.74 ± 2.18 ^a^
LIN (%)	47.71 ± 5.02 ^b^	57.5 ± 3.33 ^a^	55.03 ± 5.35 ^a^	58.82 ± 3.78 ^a^	52.09 ± 2.85 ^ab^
WOB (%)	62.91 ± 3.20	66.92 ± 2.64	65.89 ± 5.62	67.88 ± 4.59	64.39 ± 2.15

Values are expressed as mean ± standard deviation. Different letters within a line indicate significant difference (*p* < 0.05). VCL, curvilinear velocity; VSL, straight-line velocity; VAP, average path velocity; BCF, beat-cross frequency; ALH, lateral head; STR, straightness (VSL/VAP); LIN, linearity (VSL/VCL); WOB, wobble (VAP/VCL), *n* = 5.

## Data Availability

The data presented in this study are available in the article.

## Supplementary Materials

The following supporting information can be downloaded at: https://www.mdpi.com/article/10.3390/ani12192626/s1, Figure S1: Western blotting analysis of PRODH in goat sperm, Figure S2: Effects of 2 mM proline on protein (Caspase3 and Caspase9) expression in post-thaw goat sperm.
