# Supplementary material for: Beneficial Effect of Proline Supplementation on Goat Spermatozoa Quality during Cryopreservation
Source: Animals (Basel). 2022 Sep 30;12(19):2626. doi: 10.3390/ani12192626 (PMC9558967; doi:10.3390/ani12192626)
Supplement: Supplementary file 1 [file animals-12-02626-s001.zip › animals-1929278-supplementary.pdf]

animals

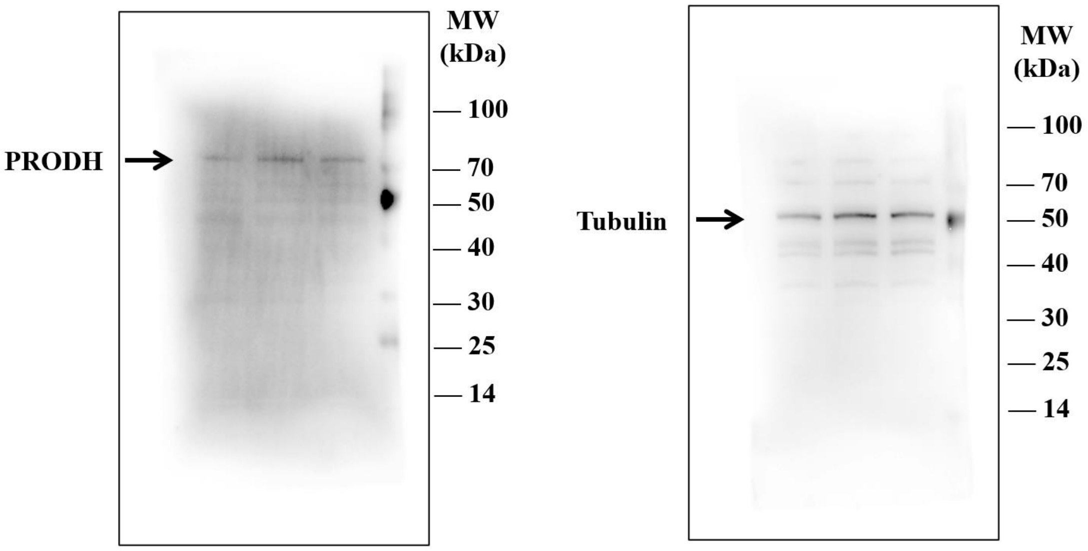

Figure S1. Western blotting analysis of PRODH in goat sperm

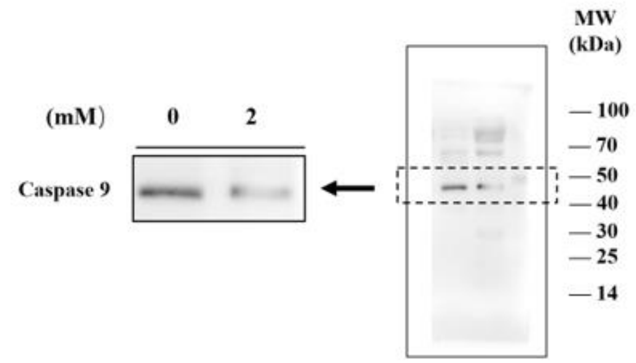
MW
(kDa)
(mM)
0
2
Caspase 9
100
70
50
40
30
25
14

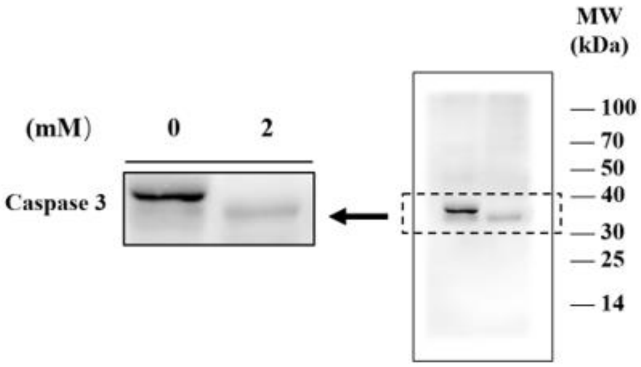
MW
(kDa)
(mM)
0
2
Caspase 3
100
70
50
40
30
25
14

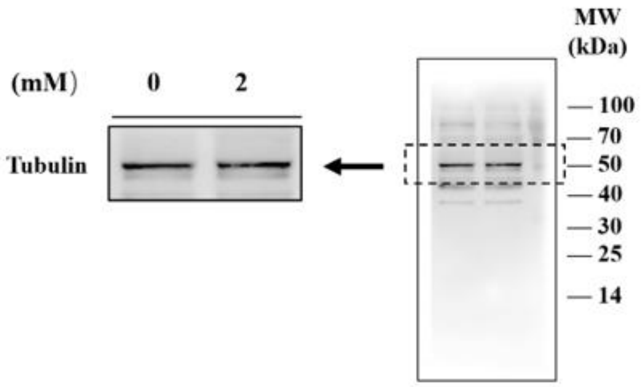
MW
(kDa)
(mM)
0
2
Tubulin
100
70
50
40
30
25
14

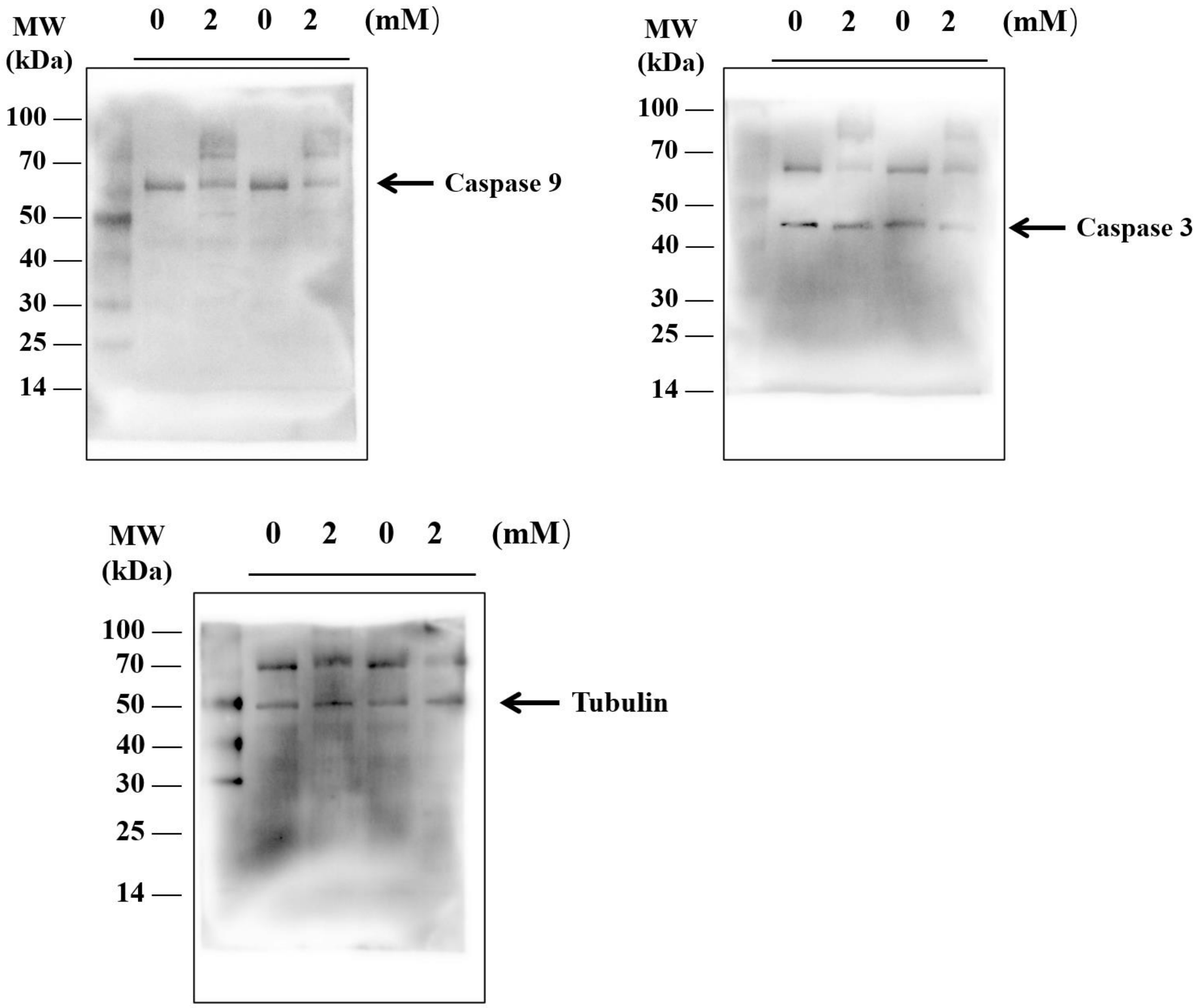

Figure S2. Effects of 2 mM proline on protein (Caspase3 and Caspase9) expression in post-thaw goat sperm.
